# Gliosis induction on locus coeruleus in a living liver donor experimental model: A brief review

**DOI:** 10.22038/IJBMS.2023.70847.15389

**Published:** 2024

**Authors:** Abril Alondra Barrientos-Bonilla, Paola Belem Pensado-Guevara, Rasajna Nadella, Aurora del Carmen Sánchez-García, Laura Mireya Zavala-Flores, Daniel Hernandez-Baltazar

**Affiliations:** 1 Centro de Investigaciones Biomédicas, Universidad Veracruzana, Xalapa, Veracruz, Mexico; 2 Instituto de Neuroetología, Universidad Veracruzana, Xalapa, Veracruz, Mexico; 3 International Collaboration (ID Proyect 1840), India; 4 Laboratorio de Neuropatología Experimental. Instituto Nacional de Neurología y Neurocirugía, CDMX, Mexico; 5 Centro de Investigación Biomédica del Noreste, IMSS. Monterrey, Nuevo León, Mexico; 6 Investigadores por México CONAHCyT-Instituto de Neuroetología. Universidad Veracruzana, Xalapa, Veracruz, Mexico

**Keywords:** Animal model, Brain, Cell damage, Immflamation, Liver, Stress, Surgery, Transplantation

## Abstract

Living Donor Liver Transplantation (LDLT) is a promising approach to treating end-stage liver diseases, however, some post-operatory complications such as pneumonia, bacteremia, urinary tract infections, and hepatic dysfunction have been reported. In murine models using partial hepatectomy (PHx), a model that emulates LDLT, it has been determined that the synthesis of hepatic cell proliferation factors that are associated with noradrenaline synthesis are produced in locus coeruleus (LC). In addition, studies have shown that PHx decreases GABA and 5-HT2A receptors, promotes loss of dendritic spines, and favors microgliosis in rat hippocampus. The GABA and serotonin-altered circuits suggest that catecholaminergic neurons such as dopamine and noradrenaline neurons, which are highly susceptible to cellular stress, can also be damaged. To understand post-transplant affections and to perform well-controlled studies it is necessary to know the potential causes that explain as a liver surgical procedure can produce brain damage. In this paper, we review several cellular processes that could induce gliosis in LC after rat PHx.

## Introduction

Liver transplantation is a treatment for end-stage liver diseases (1, 2), however, most of the organs (or their portions) coe from postmortem donors. The number of patients exceeds the number of livers available for donation. To resolve this problem, the use of liver transplantation from Living Donor Liver Transplantation (LDLT) has gained great relevance (3-5). LDLT consists of the removal and reinsertion of a fraction of the liver (16-30%) from a living, healthy, and compatible donor to a sick patient (6, 7). However, according to the Clavien-Dindo scale, postoperative complications such as keloids, hernias, bilomas, hemorrhages, pneumonia, gram-negative-related bacteremia, urinary tract infections, hepatic dysfunction, impaired regeneration, and severe ischemia/reperfusion injury (IRI) in the graft, result in small-for-size syndrome (SFSS), thrombocytopenia, and others reported in 10–30% of all cases around the world (8-10). 

At the preclinical level, the partial hepatectomy (PHx), an animal model designed by Higgins and Anderson in 1931 (11), emulates LDLT. PHx allows cutting at a maximum of 95 percent of liver parenchyma (12, 13). Studies using 30%- or 70%-PHx murine models lead to identifying proliferation, cell cycle regulation, and cell death pathways (14-16). Highlighting that to reach an efficient liver regeneration transcription factor (e.g. Nrf2, Mir33, Keap1), activation of proteins, for example, cleavage caspase-3, epidermal growth factor (EGF), transforming growth factor (TGF), tumor necrosis factor-alpha (TNF-α), humoral response factors such as interleukin 6 (IL-6), and other biomolecules such as insulin, bile acids, and noradrenaline (NA), also named norepinephrine (NE), are tightly involved (13, 17-19). 

**Figure 1:**
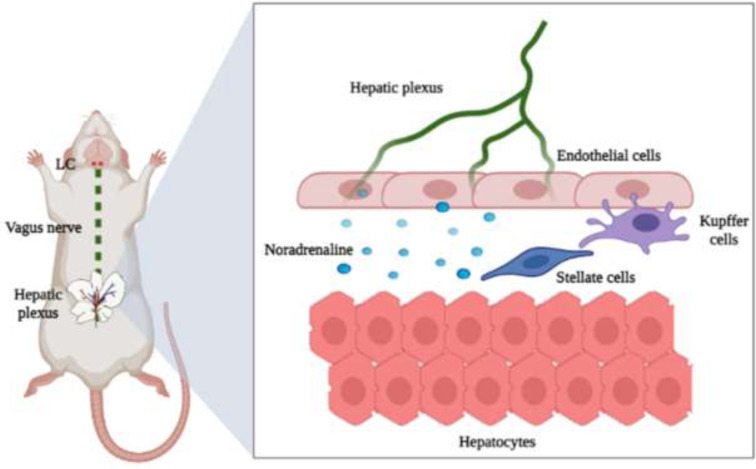
Schematic view of brain-liver axis

NA is produced on the locus coeruleus (LC) which leads to the production of growth factors that regulate the proliferation of hepatocytes (20, 21). Brain-liver communication is carried out through the hepatic plexus (Figure 1), Due to this PHx favors neuronal dysfunction due to anterograde nerve retraction (22, 23), which could produce hepatic encephalopathy or neurological abnormalities. PHx induces decreased expression of gamma-aminobutyric acid (GABA) and serotonin (5-HT2A) receptors; furthermore, PHx favors dendritic spine loss and microgliosis/astrocytosis in rat hippocampus (24-26).

***Susceptibility of noradrenergic neurons***

Stress-based susceptibility of NA neurons could favor degeneration. The clues that can explain this phenomenon are discussed in the next lines.

*First*, LC is a bilateral nucleus, is the most important noradrenergic source, and is involved in the regulation of learning, pain modulation, and among others, memory consolidation and retrieval. The morphotype-based subsets arranged in the dorsal-ventral region of the LC extend afferents to CA1 from the hippocampus and cerebellum. It has been determined that damage induced by stereotaxic manipulation in the CA1 produces hyperactivity and motor incoordination. Elevated levels of iNOS, IL-1β, and prostaglandin E2 are identified when cerebellum lose connectivity with LC, which are associated with atypical motor behavior in rats (21, 24, 27-29).

*Second*, NA is not the only neurotransmitter produced on the LC. One subset of LC neurons co-releases galanin (Gal) in the dorsal and central regions, and another subset co-releases Neuropeptide Y (NPY) in the dorsal portion. In addition, the presence of receptors for the neurotransmitter acetylcholine (nicotinic receptors, nAChRs: α3, α6, β3, and β4), GABA, orexin/hypocretin, and opioid peptides has also been identified. The fact that the same morphotypes-based subset co-releases two different neurotransmitters can compromise, in pathological conditions, their mitochondrial homeostasis, which could be a disadvantage in preventing oxidative/nitrosative imbalance (27, 30). 

*Third, *LC noradrenergic neurons can be stressed easily due to their low anti-oxidant ability. Epigenetic or morphological changes can be performed. Genomic disturbances include altered expression of genes required for the synthesis and release of NA, as well as genetic transcription errors focused on protein kinases that activate transcription factors or atypical expression of growth factors such as brain-derived neurotrophic factor (BDNF). Regarding morphology, the distress can generate aberrant neuronal arborization and loss of dendritic spines (31-33).

*Fourth*, NA participates as an inducer of the synthesis of growth factors during liver regeneration. This route requires a complementary source of NA. The alternative source of NA is the chromaffin cells of the adrenal medulla, where NA is synthesized from tyrosine, a process that is regulated by the hypothalamus and cerebral cortex. The loss of connectivity between the brain and the liver can potentiate the activation of systemic inflammation (34-36).

However, joining susceptibility neuronal plus PHx physical effects leads analyze other factors involved in the presence of gliosis in LC.

***Neuronal damage linked to PHx***

**Figure 2:**
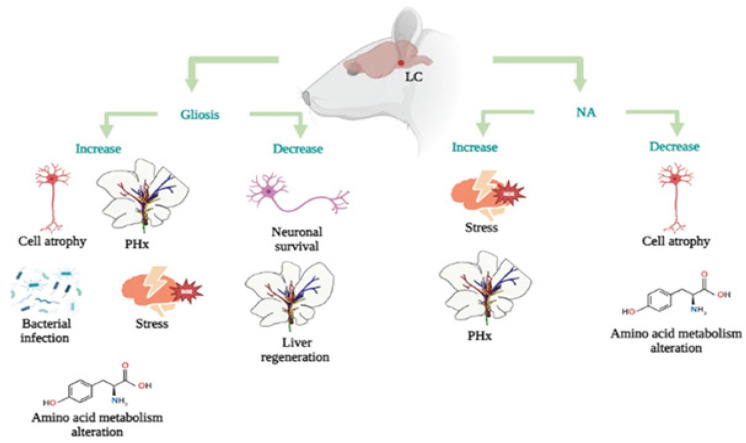
Potential risk factors to induce gliosis on LC

Morphometric changes in neurons after PHx have been described. Le *et al*. (24) reported a decrease in dendritic spines in neurons of the CA1 region and dentate gyrus from the hippocampus at 1 and 3 days post-PHx. Zhang’s group (37) identified pyknotic nuclei, nuclear membrane shrinkage, widened perinuclear space, and decreased synaptic surface area in hippocampal neurons, according to Shilpa´s laboratory which reported neuronal apoptosis after ablation of two-70%-PHx (25). To explain this fact it is necessary to revise the hot points of the physical impact of the hepatectomy procedure (Figure 2).

***Amino acid metabolism alteration***

Studies have shown that the availability of tyrosine modulates the synthesis of NA by phenylalanine hydroxylase enzyme complex. The lack of tyrosine and consequently NA due to PHx could induce distress (38, 39).

***Post-operative infection***

Lipopolysaccharide (LPS)-related endotoxemia can produce damage to the central nervous system. For example, the infection with *Lactobacillus rhamnosus* alters the GABA receptor expression on the cingulate cortex, hippocampus, amygdala, and LC (39-41). 

***Redox balance***

At the liver level it has been demonstrated that the anti-oxidant capacity remains stable during liver resection but decreases during the post-surgical period in humans, which can induce post-operative complications such as infections, keloids, or delayed liver regeneration (42-44). 

***Clinical relevance***

Neuronal dysfunction can be given in the first instance by a loss of phenotype, atrophy, and cell death mainly by apoptosis (24-26, 37). The relationship between LC and the development of neurodegenerative diseases has been studied, finding that structural and functional alterations are related to pathologies such as Alzheimer’s, Parkinson’s, and Dementia (27). In Alzheimer’s Disease, high expression of the Tau protein has been observed in LC (45-46), as well as a neural decrease of 15 to 55%, and abnormal NA content in the LC affecting memory, perception, and visuospatial ability (47); while in Parkinson’s disease and dementia there is a neuronal decrease of LC (27) followed by microgliosis and disruption in the biosynthesis of NA (48).

The vagus nerve originates from the posterior lateral sulcus of the medulla oblongata, the right and left portions of this nerve make a connection with the hepatic plexus, which is attached to the hepatic artery to integrate into the hepatic parenchyma. Artwork was performed in Biorender.

Artwork was performed in Biorender.

LC: Locus coeruleus

## Conclusion

The data support the hypothesis that a transient neuroinflammatory process on LC is activated during liver regeneration, but information regarding the neuroprotective or cytotoxic effect of glial activation on this brain nucleus is of great interest to the researchers. 

## Authors’ Contributions

AA BB, R N and D HB wrote the manuscript. PB PG, AC SG and LM ZF revised the review. All the authors read and approved the final manuscript

## Financial Disclosure

This work was partially funded by the Consejo Nacional de Ciencia y Tecnología (CONAHCyT; Project # 1840) for DH-B, FIS/IMSS/PROT/G15/1480 from Instituto Mexicano del Seguro Social for LMZ-F. AAB-B and PBP-G received scholarships # 744576 and # 797660 from CONAHCyT for postgraduate studies in biomedical sciences and neuroethology, respectively.

## Conflicts of Interest

The authors declare no conflicts of interest. 
